# Characterization of Putative Cholesterol Recognition/Interaction Amino Acid Consensus-Like Motif of *Campylobacter jejuni* Cytolethal Distending Toxin C

**DOI:** 10.1371/journal.pone.0066202

**Published:** 2013-06-06

**Authors:** Chih-Ho Lai, Cheng-Kuo Lai, Ying-Ju Lin, Chiu-Lien Hung, Chia-Han Chu, Chun-Lung Feng, Chia-Shuo Chang, Hong-Lin Su

**Affiliations:** 1 Department of Microbiology, School of Medicine, Graduate Institute of Basic Medical Science, China Medical University, Taichung, Taiwan; 2 Department of Life Sciences, Agricultural Biotechnology Center, National Chung Hsing University, Taichung, Taiwan; 3 School of Chinese Medicine, China Medical University, Taichung, Taiwan; 4 Department of Biochemistry and Molecular Medicine, University of California Davis Comprehensive Cancer Center, Sacramento, California, United States of America; 5 Biomedical Science and Engineering Center, National Tsing Hua University, Hsinchu, Taiwan; 6 Department of Internal Medicine, China Medical University Hospital, Taichung, Taiwan; 7 Department of Physical Therapy, China Medical University, Taichung, Taiwan; Iowa State University, United States of America

## Abstract

Cytolethal distending toxin (CDT) produced by *Campylobacter jejuni* comprises a heterotrimeric complex formed by CdtA, CdtB, and CdtC. Among these toxin subunits, CdtA and CdtC function as essential proteins that mediate toxin binding to cytoplasmic membranes followed by delivery of CdtB into the nucleus. The binding of CdtA/CdtC to the cell surface is mediated by cholesterol, a major component in lipid rafts. Although the putative cholesterol recognition/interaction amino acid consensus (CRAC) domain of CDT has been reported from several bacterial pathogens, the protein regions contributing to CDT binding to cholesterol in *C. jejuni* remain unclear. Here, we selected a potential CRAC-like region present in the CdtC from *C. jejuni* for analysis. Molecular modeling showed that the predicted functional domain had the shape of a hydrophobic groove, facilitating cholesterol localization to this domain. Mutation of a tyrosine residue in the CRAC-like region decreased direct binding of CdtC to cholesterol rather than toxin intermolecular interactions and led to impaired CDT intoxication. These results provide a molecular link between *C. jejuni* CdtC and membrane-lipid rafts through the CRAC-like region, which contributes to toxin recognition and interaction with cholesterol.

## Introduction

*Campylobacter jejuni* is a Gram-negative bacterium that commonly causes diarrhea in humans worldwide [1]. *C. jejuni*-associated enterocolitis is typically associated with a local acute inflammatory response that involves intestinal tissue damage [2]. Several bacterial virulence factors of *C. jejuni*, including adhesion molecules, flagella, and cytotoxins, have been investigated for their roles in host pathogenesis [3]. Although cytolethal distending toxin (CDT) from *C. jejuni* has been characterized [4], the molecular mechanisms underlying CDT involvement in *C. jejuni*-induced host pathogenesis requires further investigation.

CDT is a bacterial genotoxin consisting of a heterotrimeric complex comprising CdtA, CdtB, and CdtC [5]. CDT holotoxin, which is produced by various important Gram-negative bacteria, has been well characterized [6]. Several studies have shown that CdtA and CdtC are essential for mediating toxin binding to the cytoplasmic membrane of target cells [5], [7], [8]. Upon binding to the cell membrane, CdtB is internalized into the cells and is further translocated into the nucleus [9]. Irrespective of the bacterial species, the nuclear-translocated CdtB contains type I deoxyribonuclease activity that can cause double-strand DNA breakage (DSB) followed by cell cycle arrest at G2/M [10]. These insights into the biological function of the CDT holotoxin have identified CDT as an essential factor for *C. jejuni*-induced pathogenesis in host cells [11].

Increasing evidence has demonstrated that CdtA and CdtC form a heterodimeric complex that enhances attachment of the toxin to cell membranes [5], [7], [12]. The presence of a C-terminal non-globular structure in CdtA and CdtC is important for toxin assembly and attachment to the cell membrane [8]. Structural studies have shown that CdtA and CdtC exhibit ricin-like lectin structures [10], indicating that membrane glycoproteins contribute to the binding of CDT to cells [13]. A recent study demonstrated that both membrane carbohydrates and cholesterol play a critical role in CDT binding to cultured cells [14]. As shown by several studies, reduction in membrane cholesterol levels prevents CdtA/CdtC from binding to target cells and results in attenuated CDT intoxication [15], [16]. In previous studies on toxin interactions with cholesterol-rich microdomains, CdtC from *Aggregatibacter actinomycetemcomitans* was found to contain a cholesterol recognition/interaction amino acid consensus (CRAC) region [L/V(X)_1–5_Y(X)_1–5_R/K] that is important for toxin binding and facilitating endocytosis of CdtB [17]. These lines of evidence support the hypothesis that CdtA/CdtC might harbor a unique motif required for toxin binding to cholesterol. Although putative sequences of *C. jejuni* CdtA/CdtC required for binding to cultured cells have been reported [7], the exact protein regions contributing to toxin recognition and interaction with cholesterol have not yet been determined. Our recent study has shown that cholesterol provides a platform for *C. jejuni* CDT intoxication of cells [16]; however, the molecular mechanism for the interaction of *C. jejuni* CdtA/CdtC with cholesterol remains unknown.

In this study, we examined the potential CRAC-like region present in CdtC from *C. jejuni* and functionally assessed this candidate cholesterol-binding motif in CdtC. Mutational analysis of the CRAC-like region showed that a tyrosine residue is essential for CdtC membrane binding but not for toxin assembly. Our results further indicated that a putative CRAC-like region is present in *C. jejuni* CdtC, which contributes to the interaction with membrane cholesterol-rich microdomains and facilitates toxin intoxication.

## Materials and Methods

### Reagents and antibodies

Antibody against proliferating cell nuclear antigen (PCNA) was purchased from Santa Cruz Biotechnology (Santa Cruz, CA). Anti-actin mouse monoclonal antibody was purchased from Upstate Biotechnology (Lake Placid, NY). Alexa Fluor 488-conjugated anti-mouse IgG was purchased from Invitrogen (Carlsbad, CA). Antiserum against each CDT subunit was prepared as described previously [16]. All other chemicals, water-soluble cholesterol, and cholesterol depletion agent–methyl-β-cyclodextrin (MβCD) were purchased from Sigma-Aldrich (St. Louis, MO).

### Construction of *cdtC^Y81P^* and *cdtC^T163A·L164A^* mutants

*cdtC* ligated pET21d [16] was utilized as the template for mutagenesis. Amino acid substitution was introduced into the *cdtC* gene by site-directed mutagenesis. The forward and reverse oligonucleotide primers used for amplification of *cdtC^Y81P^* were cdtC-F (5′-GAACTTCCTTTTGGTCCTGTGCAATTTAC-3′) and cdtC-R (5′-GTAAATTGCACAGGACC AAAAGGAAGTTC-3′). The oligonucleotide primers used for generation of *cdtC^T163A·L164A^* were forward: 5′-CTTTGGAATAGCCCCTTGCGCCGCAGATCCTATTTTTT-3′ and reverse: 5′-CTTTGGAA TAGCCCCTTGCGCCGCAGATCCTATTTTTT-3′. Amplification of *cdtC* mutant was carried out by using the QuikChange II site-directed mutagenesis system (Stratagene, Santa Clara, CA). The mutation of *cdtC* was verified by DNA sequencing.

### Purification of CDT Subunits

Each recombinant His-tagged CDT subunit was cloned and prepared as previously described [16]. Briefly, *E. coli* BL21-DE3 cells harboring CdtA, CdtB, CdtC or CdtC^Y81P^ expression plasmids were induced by 0.5 mM of isopropyl β-D-thiogalactopyranoside (IPTG) at 37°C for 3 h. The expressed His-tagged CdtA, CdtB, and CdtC fusion proteins were purified by metal affinity chromatography (Clontech, Palo-Alto, CA) and assessed by SDS-PAGE and western blot.

### SDS-PAGE and Western Blot Analyses

To test the reconstitution of CDT holotoxin, each recombinant Cdt subunit (200 nM) was prepared and incubated at 37°C for 5 min allowed to assemble followed by incubation with cells [16]. CDT holotoxin-treated cells were then washed three times with PBS and boiled in SDS-PAGE sample buffer for 5 min. The samples were resolved by 12% SDS-PAGE and transferred onto polyvinylidene difluoride membranes (Millipore, Billerica, MA). The membranes were incubated with each antiserum against each CDT subunit followed by incubated with horseradish peroxidase (HRP)-conjugated secondary antibodies (Invitrogen). The proteins of interest were detected using the ECL Western Blotting Detection Reagents (GE Healthcare, Piscataway, NJ) and detected using X-ray film (Kodak, Rochester, NY).

### Structural simulation

The structure-based virtual docking of cholesterol for target protein was described previously with a slight modification [18]. To build the cavity model of *C. jejuni* CdtC, the *H. ducreyi* CdtC (Protein Data Bank Code: 1SR4 [10]) was employed as a template using homology detection tool, SWISS-MODEL [19]. The initial moiety of docked cholesterol into predicted CRAC-domain cavity was carried out using GEMDOCK [20]. Energy minimization on both the predicted CdtC model and the initial moiety were prepared by Discovery Studio v3.0 (http://accelrys.com/products/discoverystudio/). To further refine the initial docked model through molecular dynamics, the final predicted docked model was retrieved using CDOCKER with CHARMm force field [21]. Structural figures were generated with the program PyMol (http://www.pymol.org).

### Dot Blot Analysis

The binding activities of CdtC^wt^ and CdtC^Y81P^ to cholesterol were analyzed by dot blot as described previously [18]. Briefly, the polyvinylidene fluoride membranes (Millipore, Billerica, MA) were prepared, and a series concentrations of water-soluble cholesterol (0, 1.56, 3.13, 6.25, 12.5, 25, 50, 100, 200 µM) (Sigma-Aldrich) were added onto membranes at the center of grid with vacuums. The membranes were blocked by 3% BSA in PBS for 1 h followed by incubated with 200 nM CdtC^wt^ or CdtC^Y81P^ at room temperature for 2 h. The membranes were washed with PBS and probed with anti-CdtC antiserum and anti-mouse-HRP antibody (Santa Cruz) at room temperature for 1 h, respectively. The images were visualized by using Image Quant LAS-4000 (Fujifilm, Tokyo, Japan). The relative density of images was quantified by using UN-SCAN-IT software (Silk Scientific Corporation, Orem, UT).

### Cell Culture

CHO-K1 cells (Chinese hamster ovary cells, CCL-61; American Type Culture Collection, Manassas, VA) and AGS cells (human gastric adenocarcinoma cells, CRL 1739) were cultured in F12 medium (HyClone, Logan, UT). COLO205 cells (human colon adenocarcinoma cells, CCL-222) were cultured in RPMI 1640 medium (Invitrogen). All culture media were supplemented with 10% complement-inactivated fetal bovine serum (HyClone, Logan, UT) and penicillin/streptomycin (Invitrogen). The cells were maintained at 37 °C in a humid atmosphere containing 5% CO_2_.

### Cell Binding Assay

CHO-K1 cells were exposed to 200 nM CDT holotoxin or an individual CDT subunit at 4°C for 2 h. The cells were washed twice with ice-cold PBS and fixed with 1% paraformaldehyde (Sigma-Aldrich) for 30 min. The cells were washed three times, and then incubated with anti-CdtB, or anti-CdtC antisera followed by Alexa Fluor 488-conjugated anti-mouse IgG (Invitrogen). The stained cells were subjected to cell cycle analysis using an FACSCalibur flow cytometer (Becton Dickinson, San Jose, CA). The data were analyzed using Cell Quest software WinMDI (Verity Software House). All samples were examined in triplicate from three independent experiments.

### Immunofluorescence

CHO-K1 cells were plated at a density of 5×10^4^ in six-well plates and incubated for 24 h. Cells were exposed to 200 nM of CDT subunit (CdtC^wt^ or CdtC^Y81P^) or CDT holotoxin (CdtABC^wt^ or CdtABC^Y81P^, 200 nM each subunit) at 11°C. After 1 h, the cells were washed and fixed in 1% paraformaldehyde (Sigma-Aldrich) for 30 min followed by permeabilized with 0.1% Triton X-100 for 30 min. Cells were incubated with and anti-CdtB, anti-CdtC antisera and probed with Alexa Fluor 488-conjugated anti-mouse IgG (Invitrogen). The prepared samples were then observed by a confocal laser-scanning microscope (Zeiss LSM 510; Carl Zeiss, Göttingen, Germany) with a 100× objective (oil immersion; aperture, 1.3).

### Isolation of Nuclear Fraction

To explore the localization of CdtB in the nucleus of target cells, CHO-K1 cells were exposed to 200 nM CdtABC^wt^ or CdtABC^Y81P^ holotoxin at 37°C for 4 h. The nuclear proteins were isolated using a nuclear extraction kit (Pierce, Rockford, IL). All protein concentrations were determined by colorimetric assay using the Bio-Rad assay kit (Bio-Rad, Hercules, CA). The isolated proteins (30 µg) from the nuclear fractions were then subjected to western blot for analysis of CdtB localization.

### Cell Cycle Analysis

Cells were treated with CdtABC^wt^ or CdtABC^Y81P^ holotoxin for 24 h. Cells were harvested and fixed with ice-cold 70% ethanol for 2 h and stained with 20 µg/ml propidium iodide (Sigma-Aldrich) containing 1 mg/ml RNase (Sigma-Aldrich) and 0.1% Triton X-100 for 1 h. The stained cells were analyzed with an FACSCalibur flow cytometer (Becton Dickinson, San Jose, CA). The data were collected using 10,000 cells from each sample and analyzed using Cell Quest software WinMDI (Verity Software House, Topsham, ME). All samples were examined in triplicate from three independent experiments.

### Statistical Analysis

The Student's *t*-test was used to calculate the statistical significance of experimental results between two groups. A *P* value of less than 0.05 was considered statistically significant.

## Results

### Generation and Characterization of Wild-type and Mutant CDT Subunits

**Figure 1:**
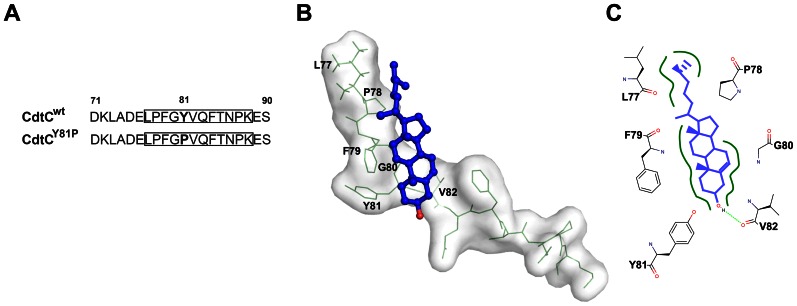
Molecular modeling of the interaction of CdtC^wt^ and cholesterol. (A) Schematic representation of a partial sequence of the CRAC-like motif in wild-type and mutant CdtC. The numbers indicate the positions of the amino acid residues. The putative CRAC-like motifs are in boxes. The amino acids in boldface indicate the residues targeted for substitution. (B) Structural model of cholesterol was in complex with putative CRAC of CdtC^wt^. The cholesterol was shown in stick and colored in blue. The number of amino acids shown in CdtC^wt^ directly interacted with the cholesterol-binding sites. Oxygen atom localized in cholesterol and amino acids were shown in red. (C) Cholesterol showed hydrophobic interactions with L77, P78, F79, G80, Y81, and V82 (boldface lines in green), and formed a hydrogen bond with V82 (dash line in green). Modeling simulation was performed using PyMol, as described in the Materials and Methods.

We recently demonstrated that CDT association with CHO-K1 cells requires intact cholesterol-rich microdomains [16]. A specific conserved sequence, the CRAC motif [L/V(X)_1–5_Y(X)_1–5_R/K], may contribute to the association of proteins with cholesterol [22]. To test this, we analyzed the amino acid sequence of CdtC, which contained a putative CRAC-like motif (^77^LPFGY^81^VQFTNPK^88^) (Fig. 1A). To assess whether this CRAC-like motif is required for CdtC binding to lipid rafts and CDT intoxication of cells, we used site-directed mutagenesis to construct a single residue-substituted mutant. The tyrosine residue that plays an important role for protein binding to cholesterol was thus replaced with a proline residue (Y81P). The mutant and wild-type CDT subunits were then subjected to SDS-PAGE (Fig. S1A) and western blot (Fig. S1B) analyses. The purity and protein expression levels of CdtC^Y81P^ were similar to those of CdtC^wt^. The integrity of the toxin complex was then assessed by western blot. As shown in Fig. S1C, both CdtC^wt^ and CdtC^Y81P^ can be assembled stably with other holotoxin elements.

### The CRAC-like Motif is Essential for CdtC Binding to Cholesterol

Structure-based virtual docking was employed to assess that cholesterol binding to a CRAC sequence with 12 amino acid residues (^77^LPFGYVQFTNPK^88^) of CdtC^wt^. Docking analysis showed that the putative CRAC-like motif created a hydrophobic groove, which enabled cholesterol to localize to it (Fig. 1B). The best favored conformations of cholesterol were found by docking and the surface represented the same helix. In addition, cholesterol was found to be bound by hydrophobic interactions with the protein residues L77, P78, F79, G80, Y81, and V82 and to form a hydrogen bond with V82 which shown within hydrogen-bonding distance of the cholesterol oxygen (2.8 Å) (Fig. 1C). The results of the molecular modeling showed that cholesterol fits into the hydrophobic groove of the CRAC-like motif.

**Figure 2:**
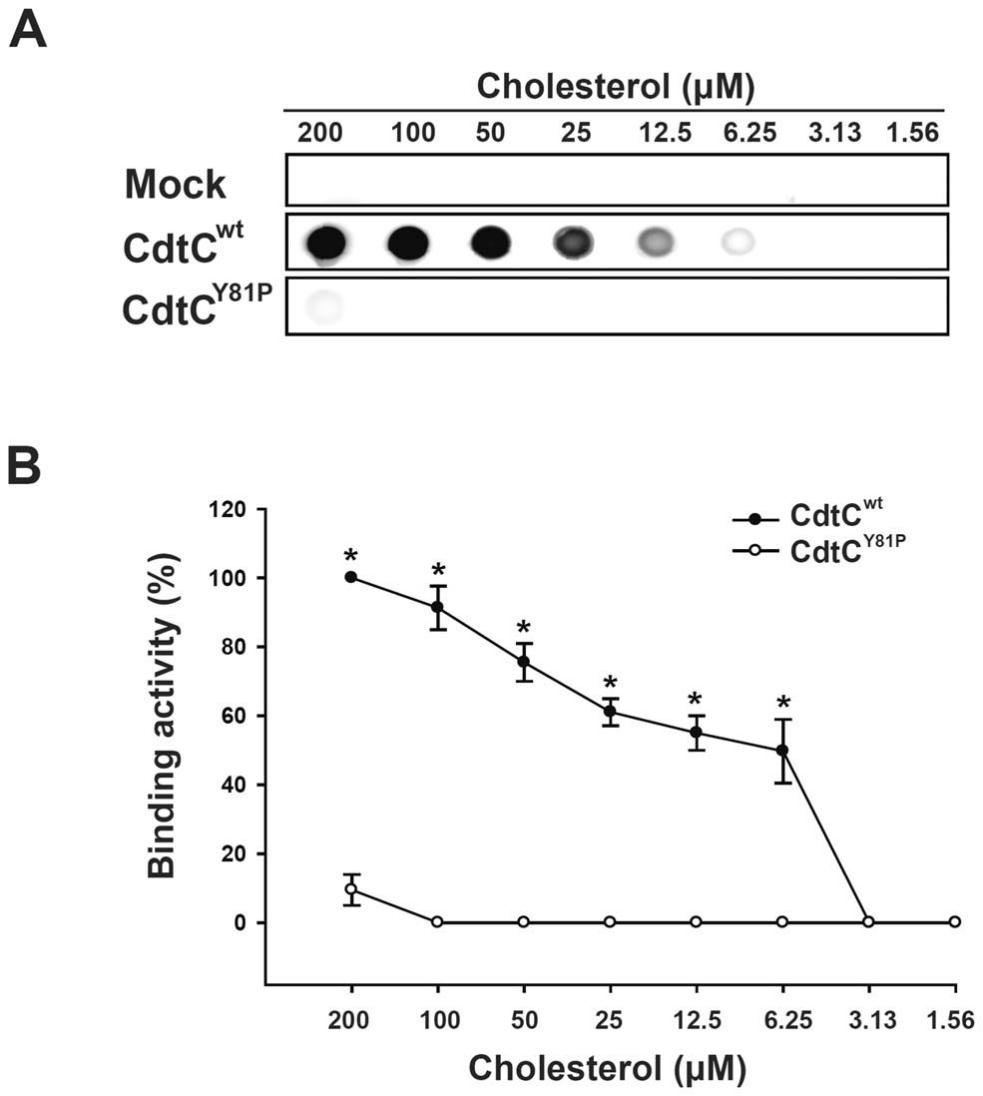
Binding of CdtC to cholesterol. (A) Direct binding of wild-type CdtC (CdtC^wt^) or mutant CdtC (CdtC^Y81P^) at various concentrations of cholesterol were analyzed by dot blot. (B) The binding activities of CdtC to cholesterol were quantified by densitometric analysis in 3 independent experiments. *, *P*<0.05 was considered as statistically significant.

To further assess whether the CRAC-like motif played a role in the CdtC-cholesterol interaction, the binding activities of CdtC^wt^ and CdtC^Y81P^ to cholesterol were analyzed by dot blot. As shown in Fig. 2A, the binding activity of CdtC^wt^ to immobilized cholesterol was concentration dependent. In contrast, direct binding to cholesterol was not detected for the CdtC^Y81P^ mutant (Fig. 2B). These results indicated that the CRAC-like motif mediates CdtC recognition and cholesterol binding.

### The CRAC-like Motif is Required for the Association of CDT with the Cell Membrane

**Figure 3:**
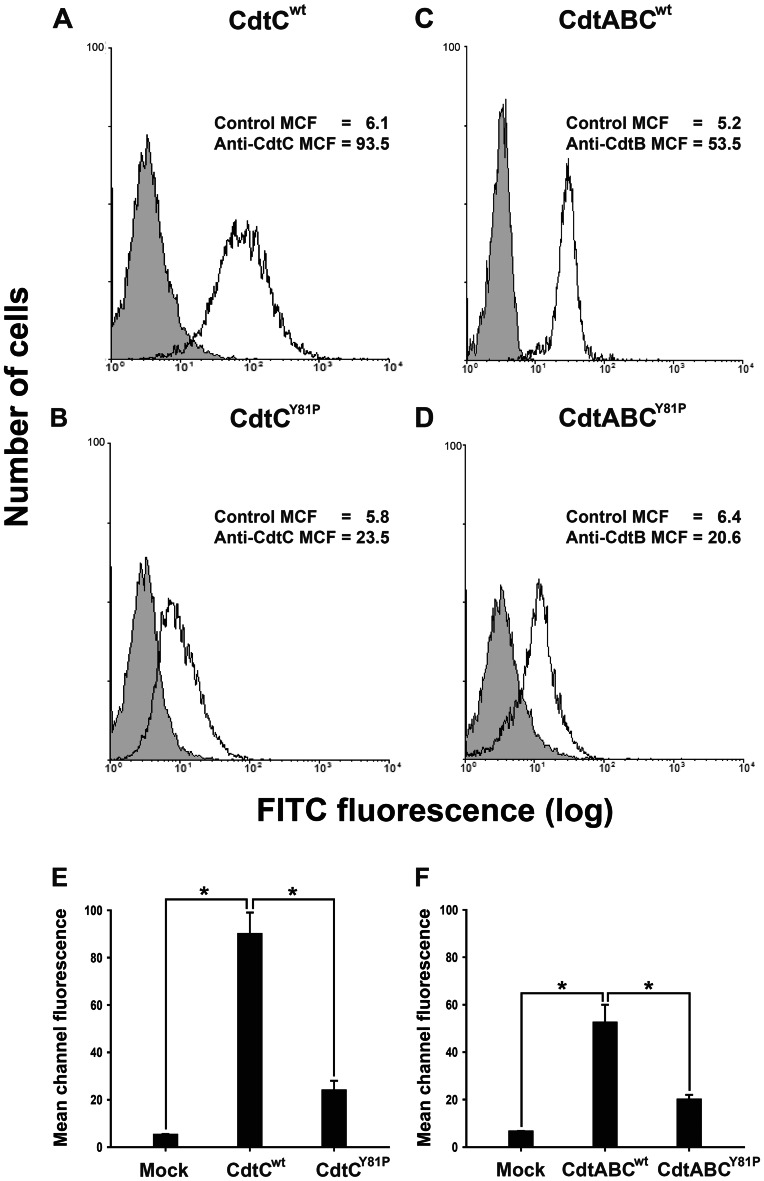
Effects of mutating the CRAC-like region on the binding of CdtC and CDT holotoxin to cells. CHO-K1 cells were treated with 200 nM of (A) CdtC^wt^, (B) CdtC^Y81P^, (C) CdtABC^wt^, or (D) CdtABC^Y81P^ at 4°C for 2 h. The cells were washed and probed with control preimmune serum (gray histograms) or antisera against CdtC (A, B) or CdtB (C, D) (white histograms), followed by staining with Alexa Fluor 488-conjugated anti-mouse IgG. The binding activity was analyzed by flow cytometry. The numbers represent the mean channel fluorescence (MCF). The quantitative data represent the mean and standard deviation values from 3 independent experiments (E, F). The asterisks indicate statistical significance (*P<0.05).

We then analyzed whether the CRAC-like motif is important for the association of CDT subunits with cell membranes. CHO-K1 cells were incubated with CdtC^wt^ or CdtC^Y81P^ for 2 h at 4°C and were analyzed by flow cytometry for the presence of CDT subunits on the cell membrane. As shown in Fig. 3A, CdtC^wt^ was associated with the cell membrane, and the MCF for anti-CdtC was 93.5. However, upon exposure of cells to CdtC^Y81P^, the MCF for anti-CdtC reduced to 23.5 (Fig. 3B). We further tested whether the CRAC-like motif mutant could affect the binding of holotoxin to cells. The levels of MCF for anti-CdtB were 53.5 and 20.6 when the cells were exposed to CdtABC^wt^ and CdtABC^Y81P^, respectively (Fig. 3C, D). Notably, compared with the binding activities of CdtC^wt^ and CdtABC^wt^, the binding activities of both CdtC^Y81P^ and CdtABC^Y81P^ to cell membranes were significantly lower (Fig. 3E, F).

**Figure 4:**
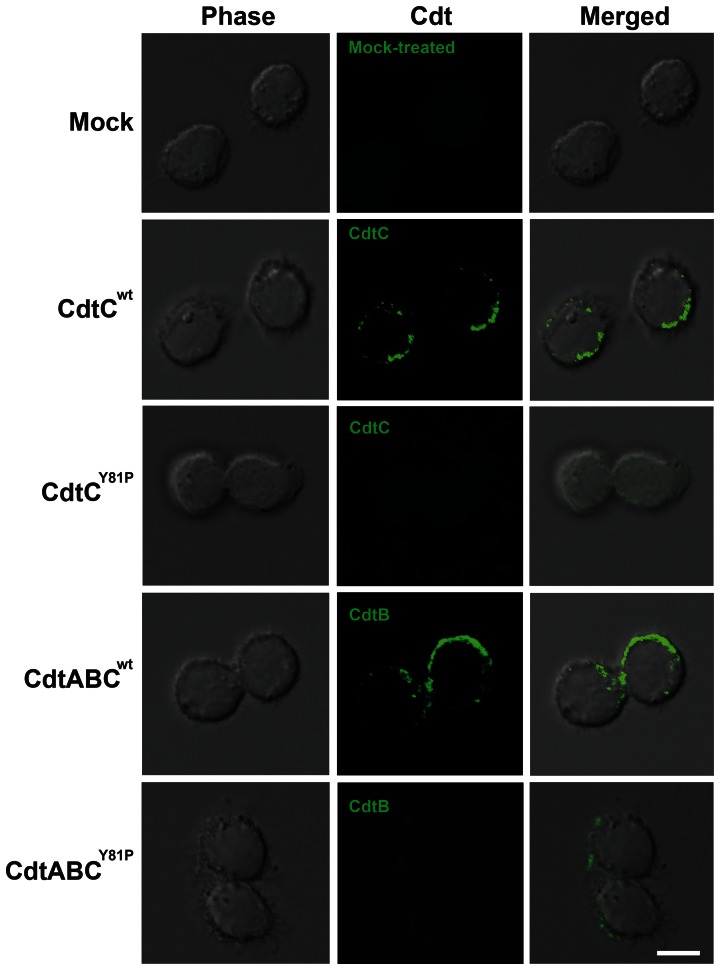
The role of the CRAC-like region in the association of CdtC and holotoxin with cells. CHO-K1 cells were incubated with mock medium alone or with 200 nM of CdtC^wt^, CdtC^Y81P^, CdtABC^wt^, or CdtABC^Y81P^ at 11°C for 1 h. The cells were probed with control preimmune serum (mock) or antisera against CdtC (2^nd^ and 3^rd^ rows) or CdtB (4^th^ and 5^th^ rows), followed by staining with Alexa Fluor 488-conjugated anti-mouse IgG, and then analyzed by confocal microscopy. Bar, 10 µm.

We used confocal microscopy to examine whether the binding of CDT to cells was dependent on the CRAC-like motif in CdtC. The cells were treated with CdtC (CdtC^wt^ or CdtC^Y81P^) or holotoxin (CdtABC^wt^ or CdtABC^Y81P^), followed by probing with preimmune serum and antisera against CdtB or CdtC. No signal for CDT was detected in untreated cells (Fig. 4, first row), whereas CdtC^wt^ (green) apparently localized to the area around the plasma membrane (Fig. 4, second row). In cells treated with CdtABC^wt^, membrane distribution of CdtB was evident (Fig. 4, fourth row), which was similar to that of cells treated with CdtC^wt^ alone. However, the intensity of detectable fluorescence for CdtC and CdtB on the plasma membrane decreased when cells were treated with CdtC^Y81P^ or CdtABC^Y81P^ (Fig. 4, third and fifth rows). These results support our findings of CDT binding activity determined by flow cytometry (Fig. 3), indicating that the CRAC-like motif is critical for CdtC association with cells for CDT intoxication of the cells.

### Nuclear Delivery of CdtB Decreases in Cells Treated with CdtABC^Y81P^

**Figure 5:**
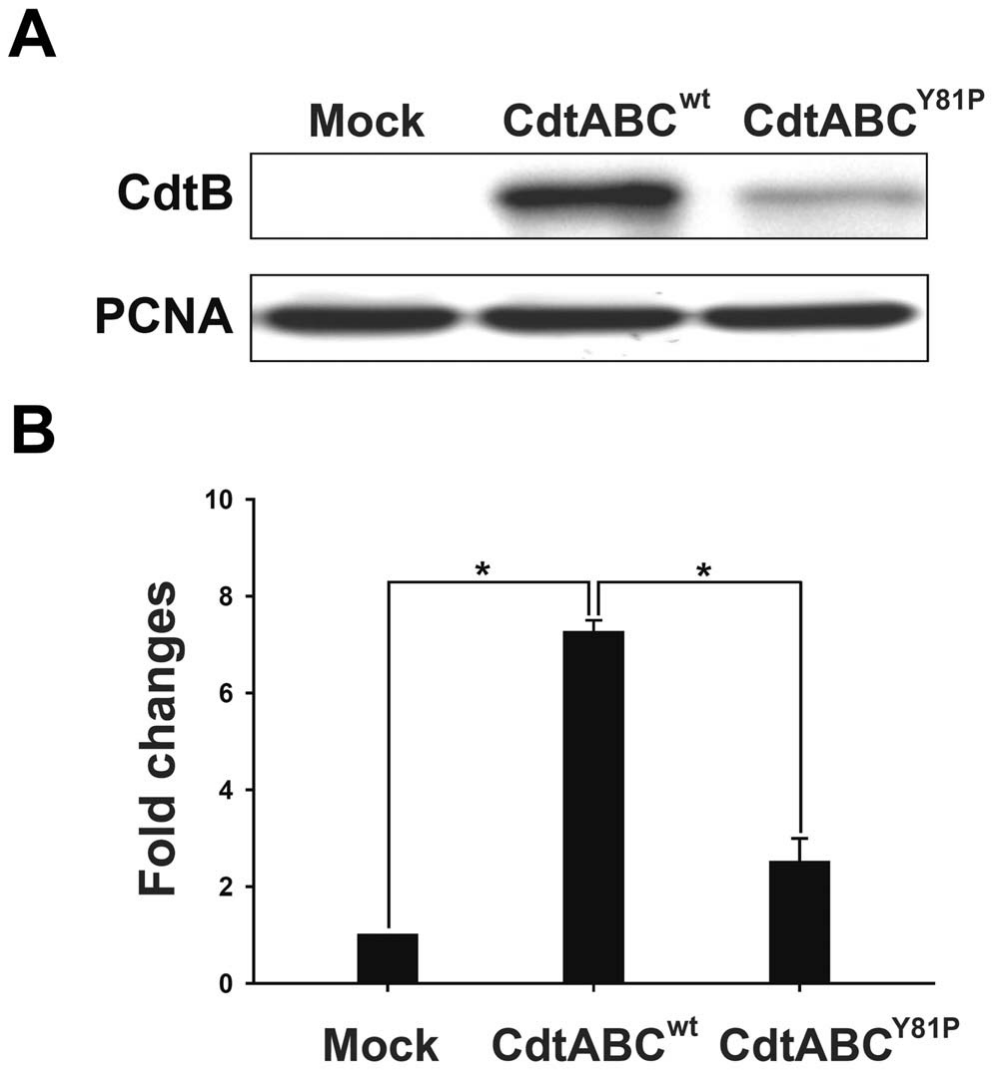
Effects of mutations in the CRAC-like motif of CdtC on CdtB nuclear localization. CHO-K1 cells were treated with mock medium alone or with CdtABC^wt^ or CdtABC^Y81P^ (200 nM each subunit) at 37°C for 4 h. (A) Nuclear fractions were prepared and subjected to western blot for analysis of CdtB. Proliferating cell nuclear antigen (PCNA) was used as a loading control for the nuclear fraction of cell lysates. (B) Expression levels of CdtB protein were analyzed using scanning densitometry. The signals of CdtB/PCNA were expressed as relative values. Statistical significance was evaluated using Student's *t*-test. *, *P*<0.05 was considered as statistically significant.

We then examined whether the nuclear localization of CdtB was dependent on the CRAC-like motif present in CdtC. The cells were incubated with CdtABC^wt^ or CdtABC^Y81P^ and subjected to western blot analysis. As shown in Fig. 5, the nuclear localization of CdtB dramatically decreased in cells treated with CdtABC^Y81P^ when compared to the localization in cells treated with CdtABC^wt^. These data suggested that CdtC associates with cell membranes through the CRAC-like motif and that this association is important for the delivery of CdtB into the nucleus.

### Mutation of the CRAC-like Motif in CdtC Attenuates CDT Intoxication of Cells

**Figure 6:**
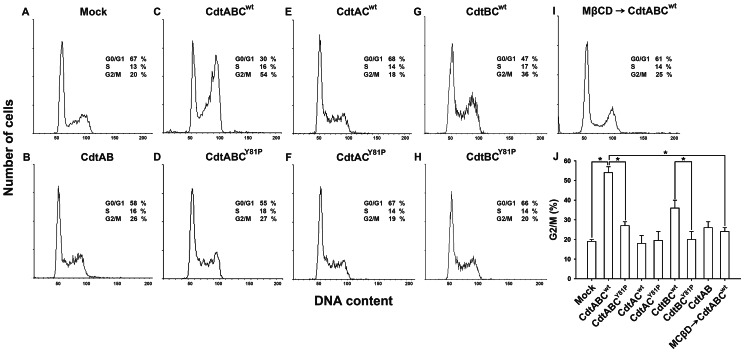
Attenuation of G2/M arrest by CdtABC^Y81P^. CHO-K1 cells were incubated with (A) mock medium alone, 200 nM of (B) CdtAB, (C) CdtABC^wt^, (D) CdtABC^Y81P^, (E) CdtAC^wt^, (F) CdtAC^Y81P^, (G) CdtBC^wt^, or (H) CdtBC^Y81P^ for 24 h, or (I) pretreated with 10 mM MβCD for 1 h followed by incubation with 200 nM CdtABC^wt^ for 24 h. The cells were stained with propidium iodide, and cell cycle distribution analyzed by flow cytometry. The proportions of cells in the G0/G1, S, and G2/M phases of the cell cycle are shown at the right of each histogram. (J) The percentage of cells in G2/M were calculated and plotted as intensity histograms. The results represent mean and standard deviation values from 3 independent experiments. *, *P*<0.05 was considered as statistically significant.

To determine whether the CRAC-like motif was required for CDT intoxication of cells, we used flow cytometry to assess cell cycle distributions. In the presence of CdtABC^wt^, 54% of cells were arrested in G2/M (Fig. 6C). However, this cell cycle arrest was attenuated upon treatment of cells with CdtABC^Y81P^ (Fig. 6D). The cell cycle distributions were not changed in cells exposed to CdtAC^wt^ and CdtAC^Y81P^, since the treatment was not contained the toxin activity subunit–CdtB (Fig. 6E, F). In contrast, the cell cycle arrest was significantly reduced in cells treated with CdtBC^Y81P^ when compared to cells treated with CdtBC^wt^ (Fig. 6G, H). Pretreating cells with MβCD followed by exposure to CdtABC^wt^ dramatically decreased the proportion of cells arrested in G2/M (Fig. 6I). These results supported the notion that the CRAC-like motif contributes to the association of CdtC with membrane cholesterol, leading to the intoxication of CDT in target cells.

**Figure 7:**
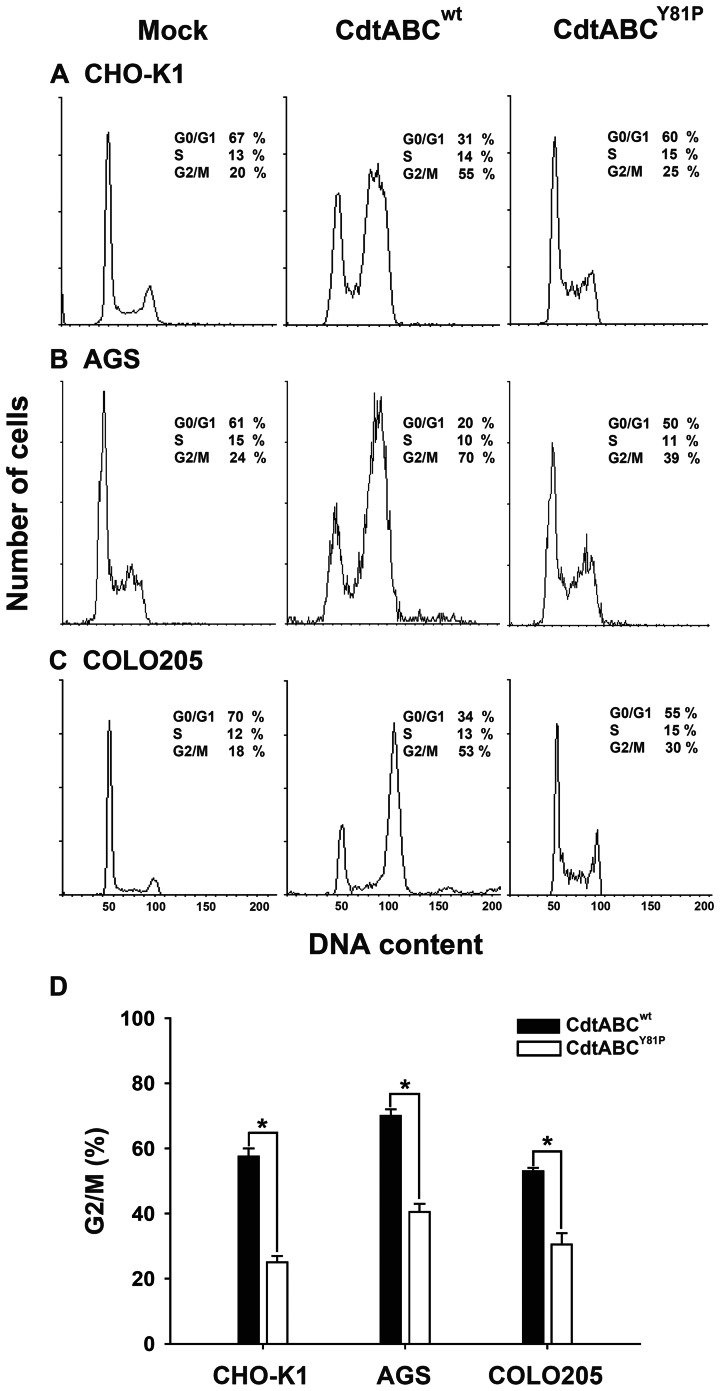
The role of the CRAC-like region in CDT intoxication of cells. Cells from the indicated lines (A) CHO-K1, (B) AGS, and (C) COLO205 were treated with mock medium, CdtABC^wt^, or CdtABC^Y81P^ (200 nM each subunit) at 37°C for 24 h. Cell cycle distribution was assessed using flow cytometry. (D) The percentage of cells in G2/M were calculated and plotted as intensity histograms. The results represent 3 independent experiments. The statistical significance of the difference was analyzed using Student's *t*-test (*P<0.05).

We next investigated whether mutation of the CRAC-like motif affected CDT-arrested cell cycles in other cell types. Two gastrointestinal cell lines (AGS and COLO205 cells) were used in this experiment. Cells were mock treated or treated with CDT holotoxin (CdtABC^wt^ or CdtABC^Y81P^) and analyzed for the cell cycle stage. As shown in Fig. 7, the proportion of cells accumulating in G2/M in the 3 lines treated with CdtABC^Y81P^ was significantly lower than that in the cells treated with CdtABC^wt^. These results again demonstrated that the CRAC-like motif present in CdtC is required for the association of CDT holotoxin with cell membranes as well as for the intoxication of target cells.

## Discussion

Lipid rafts are membrane microdomains that contained mainly cholesterol, sphingolipids, and phospholipids [23]. Membrane rafts serve as a platform for several bacterial toxins binding to target cells [24], [25], [26], [27], [28]. The most relevant example is vacuolating cytotoxin A (VacA), one of the major virulence factors secreted by *Helicobacter pylori*, which was demonstrated to exploit cholesterol-rich microdomains for its assembly on cell membranes and delivery into target cells [29]. Similarly, CDT produced by *Haemophilus ducreyi* or *A. actinomycetemcomitans* was found to interact with lipid rafts [15], [30]. In agreement with these observations, our recent study has shown that CDT from *C. jejuni* is associated with lipid rafts [16]. These lines of evidence support the hypothesis that cholesterol-rich microdomains may play a critical role in bacterial toxin assembly on cell membranes and intracellular delivery, and that these domains therefore amplify the signaling required for intoxication [31].

Indeed, not all proteins that bind cholesterol harbor CRAC-domains. For instance, the cholesterol-dependent cytolysin family of toxins contains 2 amino acids (threonine and leucine) that are responsible for interacting with cholesterol [32]. At the beginning of this investigation, we first assessed whether the threonine-leucine pair mediated the binding of CdtC to membrane cholesterol. Our results show that mutations at the threonine (T163) and leucine (L164) residues (CdtABC^T163A·L164A^) did not affect CDT intoxication, as compared to treatment with CdtABC^wt^ (Figure S2), suggesting that this pair of amino acids does not mediate the CdtC-cholesterol interaction.

In this study, the virtual docking simulation showed that *C. jejuni* CdtC contains a CRAC-like motif (Fig. 1B). The 12 amino acid residues LPFGYVQFTNPK created a hydrophobic groove that provided for hydrophobic interactions and hydrogen bonding with cholesterol. In addition, mutation of this domain decreased the cell-binding activity of CdtC. However, comparison with CdtC^wt^ showed that the interaction of CdtC^Y81P^ with CdtA and CdtB in complex formation was not altered (Fig. S1C). This finding is due to the fact that the CRAC-like domain does not extend to the CdtC N- and C-terminal regions that contribute to the interaction of CdtC with both CdtA and CdtB [8]. Taken together, our findings have demonstrated that the CRAC-like region involved in CdtC plays a critical role in toxin binding to membrane cholesterol and not in intermolecular interactions between toxin subunits.

Our results for the dot blot analysis showed that CdtC^Y81P^ did not bind to immobilized cholesterol (Fig. 2A). In addition, our data further demonstrated that cell cycle arrest was significantly lower in cells treated with CdtABC^Y81P^ or CdtBC^Y81P^ than in cells treated with CdtABC^wt^ or CdtBC^wt^ (Fig. 6). These results are supported by the functional analysis of CdtC from *A. actinomycetemcomitans* [33] and *Haemophilus parasuis* [34], indicating that CdtC contains a CRAC-like region that is important for cholesterol binding. However, the binding activity of CdtC^Y81P^ to the cell surface did not completely abolished, as shown by our flow cytometry and confocal microscopy analyses (Fig. 3 and 4), which suggests that CdtC binding to cell membranes is mediated not only by cholesterol but also by other candidate receptors. Previous studies on CDTs from *E. coli* and *A. actinomycetemcomitans* indicated a critical role of membrane carbohydrates in toxin interactions [13], [35]. In addition, structure-based analysis has shown that CdtA and CdtC from *A. actinomycetemcomitans* have similar structures comprising 3 sets of beta-sheets that are homologous to the B-chain of ricin and contain lectin repeats, indicating that carbohydrates may serve as receptors for CDT [36]. However, a recent study on cell intoxication by CDTs, which were isolated from several bacterial species, demonstrated that glycolipids are not required for CDT intoxication [14]. Although these reports indicate a discrepant role for carbohydrates in membrane association of CDTs, our results are supported by several lines of evidence indicating that cholesterol is one of the candidate receptors for *C. jejuni* CDT interactions. Further investigations are required to identify the specific receptor(s) that mediate membrane association with *C. jejuni* CDT.

Since the CDTs from different bacterial species have distinct binding activities, they are thought to have divergent target-cell preferences [14]. Although these CDTs originate from different pathogens, hijacking of cholesterol-rich microdomains for toxin function appears to be the universal mechanism underlying CDT action [15], [16], [30]. In this study, we have demonstrated that CdtC from *C. jejuni* contains a CRAC-like region that contributes to the CdtC interaction with cholesterol. Furthermore, the mutation of a tyrosine residue in the CRAC-like region impairs CdtC binding to and inhibits its intoxication of target cells. Elucidation of the target receptor for *C. jejuni* CDT would lead to better understanding of the molecular mechanisms underlying bacterial pathogenesis in host cells. The results from this study also shed light on the discovery of a novel strategy for specifically inhibiting this toxin.

## Supporting Information

Figure S1 **Characterization of wild-type and mutant CDT subunits from** ***C. jejuni*** **.** (A) Each CDT subunit (2 µg/ml) was analyzed by SDS-PAGE. (B) Western blot analysis of each CDT subunit detected via antisera against CdtA, CdtB, or CdtC. Molecular mass markers (kDa) are shown on the left. (C) *In vitro* assay for the toxin assembly of CdtABC^wt^ and CdtABC^Y81P^ at 25°C for 1 h, determined by western blot.
(TIF)

Figure S2 **Threonine (T163) and leucine (L164) of CdtC are not required for CDT intoxication.** CHO-K1 cells were treated with (A) mock medium alone, (B) CdtABC^wt^, and (C) CdtABC^T163A·L164A^ (200 nM each subunit) for 24 h. The treated cells were stained with propidium iodide, and the cell cycle distribution was analyzed by flow cytometry. The proportions of cells in the G0/G1, S, and G2/M phases of the cell cycle are shown at the right of each histogram.
(TIF)
