# Supplementary figures and images for: Characterization of Putative Cholesterol Recognition/Interaction Amino Acid Consensus-Like Motif of Campylobacter jejuni Cytolethal Distending Toxin C
Source: PLoS One. 2013 Jun 6;8(6):e66202. doi: 10.1371/journal.pone.0066202 (PMC3675143; doi:10.1371/journal.pone.0066202)

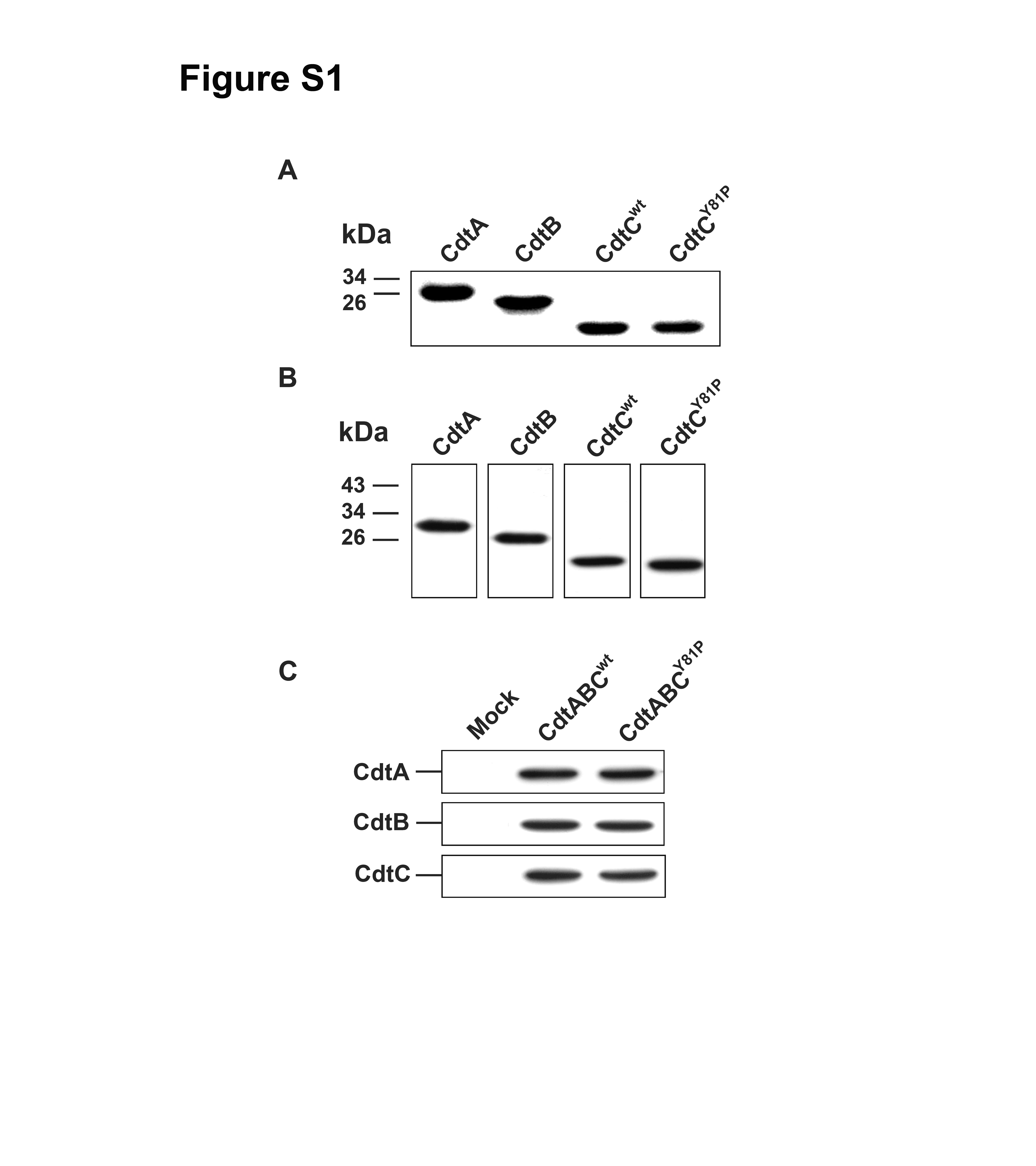

Supplement: Figure S1 — Characterization of wild-type and mutant CDT subunits from C. jejuni . (A) Each CDT subunit (2 µg/ml) was analyzed by SDS-PAGE. (B) Western blot analysis of each CDT subunit detected via antisera against CdtA, CdtB, or CdtC. Molecular mass markers (kDa) are shown on the left. (C) In vitro assay for the toxin assembly of CdtABCwt and CdtABCY81P at 25°C for 1 h, determined by western blot. (TIF) [file pone.0066202.s001.tif]

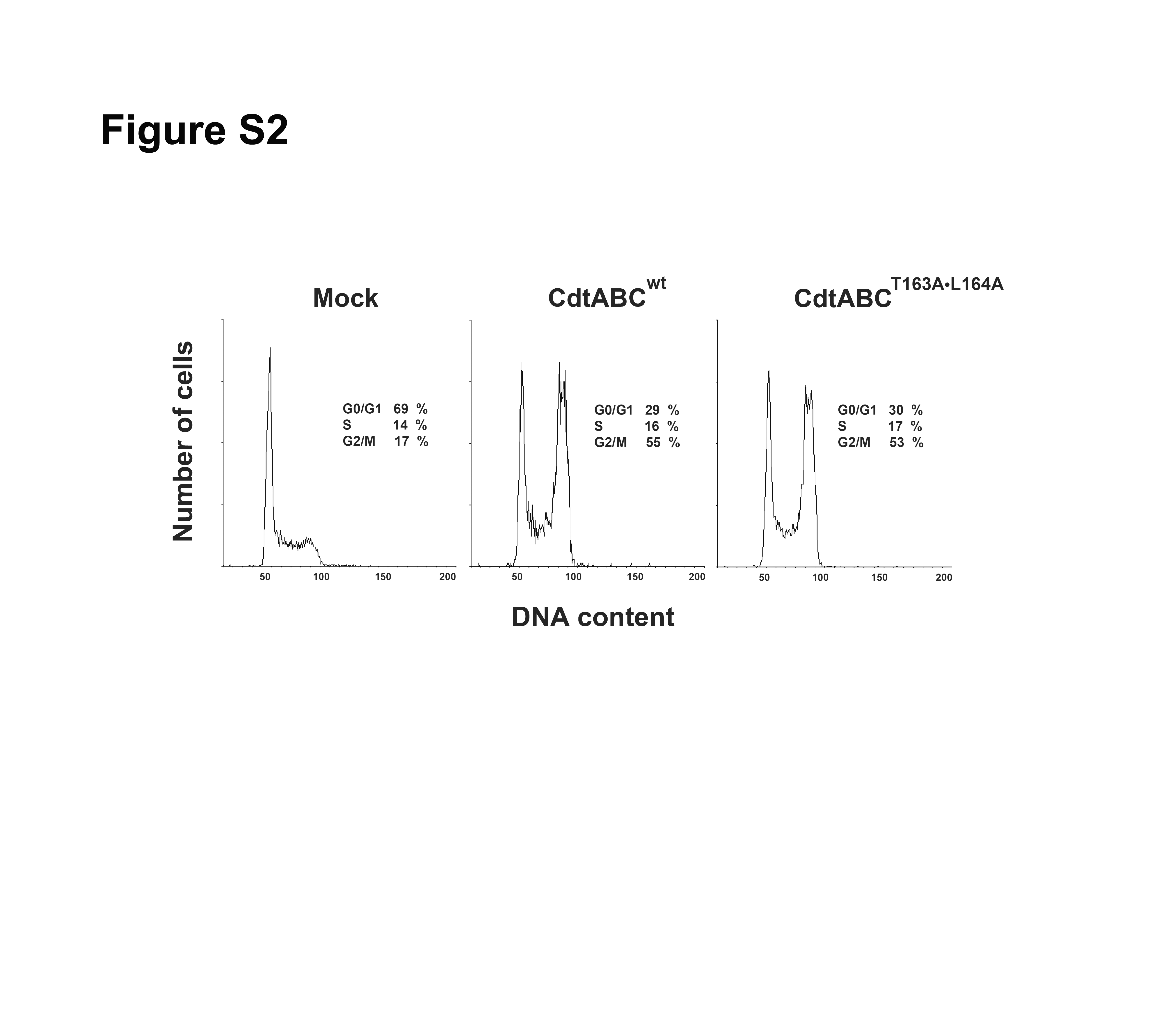

Supplement: Figure S2 — Threonine (T163) and leucine (L164) of CdtC are not required for CDT intoxication. CHO-K1 cells were treated with (A) mock medium alone, (B) CdtABCwt, and (C) CdtABCT163A·L164A (200 nM each subunit) for 24 h. The treated cells were stained with propidium iodide, and the cell cycle distribution was analyzed by flow cytometry. The proportions of cells in the G0/G1, S, and G2/M phases of the cell cycle are shown at the right of each histogram. (TIF) [file pone.0066202.s002.tif]
